# Use of artificial intelligence methods for classifying diabetic patients with polyneuropathy

**DOI:** 10.1186/1758-5996-7-S1-A4

**Published:** 2015-11-11

**Authors:** Aline Arcanjo Gomes, Eneida Yuri Suda, Cristina Dallemole Sartor, Neli Regina Siqueira Ortega, Ricky Watari, Vincent Vigneron, Isabel CN Sacco

**Affiliations:** 1 Universidade de São Paulo, Caieiras, Brazil

## Background

Diabetic polyneuropathy (DPN) has an insidious and non-homogeneous installation making it difficult to determine its onset. Therapeutic and preventive actions should target patients depending on their DPN severity status. Methods for supporting the decision making process of classifying patients can improve early health actions.

## Objective

Analyze the use of 2 artificial intelligence methods for classifying the DPN severity degree: (a) fuzzy modeling and (b) multiple correspondence analysis (MCA) and Kohonen map.

## Materials and methods

Retrospective analysis of 195 patients. The fuzzy model determined a DPN degree score (0-10) by the combination of fuzzy sets derived from clinical variables (sensorial modalities and a set of DPN-related symptoms), using if-then rules to combine the inputs with the output sets (Mamdani process), with membership functions determined by a team of 4 DPN specialists. The MCA method grouped 16 DPN-related categorical variables [sensorial modalities, symptoms, foot inspection characteristics] into micro and macro-classes (groups) after the algorithm learned the grouping pattern of the variables in the patients' cohort. A Kohonen map was used to better represent the clusters of variables that could identify different DPN severities.

## Results

Loss of tactile and vibration perceptions were decisive for classification of DPN severity using the fuzzy system, and its sensitivity and specificity in discriminating patients with and without DPN was very high (ROC=0.985). The MCA and Kohonen map identified 4 macro-classes of variables: (1) DPN absence, (2 and 3) intermediate status (2. characterized by DPN-symptoms, 3- by vibration perception reduction), and (4) severe (absence of vibration perception, foot deformities, amputation or ulcer, absence of tactile perception).

## Conclusion

The fuzzy model contributes to the early detection of DPN using typical clinical variables, and although this method strongly relies on the specialist subjectivity, it is very reliable. Software for classifying DPN severity using this Fuzzy model is available and can be easily implemented in any clinical setting as a decision support system[1]. The MCA analysis showed that tactile loss and most of the symptoms do not discriminate between DPN severity status, but the vibration perception was the most discriminative variable. Both methods are useful to help clinical decisions and DPN early detection.
